# Extinction of Yellow Fever

**Published:** 1889-02

**Authors:** 


### EXTINCTION OF YELLOW FEVER.

Blaine, the "plumed knight, the man from Maine," the incoming Secretary of State at Washington, is no "Doctor;" that is, he is is not an M. D.; but he has taught some things pretty well, notwithstanding. It is a singular thing that the most rational plan for the total extinction of yellow fever that has ever been proposed, should come from a layman. In an interview lately with a newspaper correspondent, he said that the acquisition of Cuba by the United States is a necessity, from several standpoints, and that it would be cheap at any price. Amongst other reasons, he gave, is that by improved sanitation in that home of pestilence, the United States government could stamp out yellow fever. The cost of one epidemic in the valley of the Mississippi, he said, would exceed the price paid for the Island, even though it empty the United States vaults of the accumulated surplus piled up by a Republican administration, and this is very true. There is here thrown out much food for thought. The purchase of Cuba would solve several vexing problems; one of which is "what shall we do with it" (the accumulated stealings, for such are the infamous cotton-tax and other measures of robbery to which the Southern people, especially, were subjected under twenty-five years of Republican rule).

